# Mitochondrial oxidative function in NAFLD: Friend or foe?

**DOI:** 10.1016/j.molmet.2020.101134

**Published:** 2020-12-01

**Authors:** Michael Shum, Jennifer Ngo, Orian S. Shirihai, Marc Liesa

**Affiliations:** 1 Department of Medicine, Division of Endocrinology, David Geffen School of Medicine at UCLA, 650 Charles E. Young Dr., Los Angeles, CA, 90095, USA; 2 Department of Molecular and Medical Pharmacology, David Geffen School of Medicine at UCLA, 650 Charles E. Young Dr., Los Angeles, CA, 90095, USA; 3 Molecular Biology Institute at UCLA, Los Angeles, CA, 90095, USA; 4 Department of Chemistry and Biochemistry, UCLA, Los Angeles, CA, 90024, USA

**Keywords:** Mitochondria, Mitochondrial heterogeneity, NAFLD, NASH, Lipid metabolism, H_2_O_2_, Mitophagy

## Abstract

**Background:**

Mitochondrial oxidative function plays a key role in the development of non-alcoholic fatty liver disease (NAFLD) and insulin resistance (IR). Recent studies reported that fatty liver might not be a result of decreased mitochondrial fat oxidation caused by mitochondrial damage. Rather, NAFLD and IR induce an elevation in mitochondrial function that covers the increased demand for carbon intermediates and ATP caused by elevated lipogenesis and gluconeogenesis. Furthermore, mitochondria play a role in regulating hepatic insulin sensitivity and lipogenesis by modulating redox-sensitive signaling pathways.

**Scope of review:**

We review the contradictory studies indicating that NAFLD and hyperglycemia can either increase or decrease mitochondrial oxidative capacity in the liver. We summarize mechanisms regulating mitochondrial heterogeneity inside the same cell and discuss how these mechanisms may determine the role of mitochondria in NAFLD. We further discuss the role of endogenous antioxidants in controlling mitochondrial H_2_O_2_ release and redox-mediated signaling. We describe the emerging concept that the subcellular location of cellular antioxidants is a key determinant of their effects on NAFLD.

**Major conclusions:**

The balance of fat oxidation versus accumulation depends on mitochondrial fuel preference rather than ATP-synthesizing respiration. As such, therapies targeting fuel preference might be more suitable for treating NAFLD. Similarly, suppressing maladaptive antioxidants, rather than interfering with physiological mitochondrial H_2_O_2_-mediated signaling, may allow the maintenance of intact hepatic insulin signaling in NAFLD. Exploration of the subcellular compartmentalization of different antioxidant systems and the unique functions of specific mitochondrial subpopulations may offer new intervention points to treat NAFLD.

## Highlights

• Mitochondrial function has been reported to be increased or decreased in NAFLD.
• Functionally independent subpopulations of mitochondria can clarify the conundrum of these conflicting reports.
• Maladaptive antioxidants decreasing mitochondrial H_2_O_2_ and promoting NAFLD are discussed.
• Therapies targeting mitochondria to treat NAFLD are discussed.

## 1 Overview of mitochondrial oxidative function in the liver in health and NAFLD

Despite constituting ∼2–4% of total body weight, the liver is responsible for 15% of organismal oxygen consumption in humans [1]. This large ratio of respiration/liver weight demonstrates that hepatocytes are highly enriched with mitochondria consuming oxygen to produce ATP. Accordingly, 60% of the organismal mitochondrial ATP demand imposed by glucose production and ureagenesis is covered by hepatocytes, with the remaining 40% covered by the kidney [1]. Thus, the liver is the main organ providing glucose and ketone bodies to other tissues when nutrients are scarce. During fasting, fatty acids released from adipose tissue are oxidized by hepatocyte mitochondria, generating ketones and supplying ATP that fuels gluconeogenesis and ureagenesis. It is estimated that fasting increases mitochondrial fat oxidation 10-fold [2]. Urea is produced inside mitochondria from ammonia generated by hepatic amino-acid catabolism, which is also upregulated during fasting as an additional source of ATP and carbons for gluconeogenesis.

In the fed state, hepatocytes conserve and store nutrients to fulfill their specialized function of providing nutrients to other tissues when food is scarce. To this end, insulin action in hepatocytes increases glucose storage as glycogen and transforms dietary glucose into lipids. Carbon intermediates required for lipid synthesis and ATP needed to energize anabolism are provided by mitochondria. Specifically, glucose-derived pyruvate oxidation in mitochondria elevates citrate synthesis. Citrate is then consumed to generate the carbon precursor of de novo synthesized fatty acids, namely malonyl-CoA. These new fatty acids are esterified into triglycerides and packed into lipid droplets and lipoproteins. Malonyl-CoA also promotes lipoprotein and lipid droplet expansion by blocking the entry of fatty acids into mitochondria to prevent their oxidation [2]. Importantly, large intrahepatic lipid droplets visualized by histology are not observed in healthy livers and hepatocytes can re-uptake secreted lipoproteins. Thus, lipid storage units inside healthy hepatocytes are highly dynamic, with this dynamism being an ATP-demanding process. Overall, mitochondrial oxidative function is highly active in the fed and fasted states, as mitochondria participate both in the production/storage and the consumption of glucose and lipids.

Fatty liver or simple steatosis is defined as the visualization of large lipid droplets in hepatocytes by histology. Simple steatosis is the first stage of NAFLD, which can progress to non-alcoholic steatohepatitis (NASH) characterized by liver inflammation, increased hepatocyte ballooning/swelling, cell death, and some degree of fibrosis. The pro-inflammatory NASH state can eventually lead to cirrhosis and hepatocarcinoma.

Fatty liver can occur when glucose and fatty acid availability exceeds both the capacity of adipose tissue to store energy and the demand for ATP of hepatocytes. Accordingly, steatosis is not exclusively generated by the positive energy balance associated with diet-induced obesity or overeating. Lipodystrophy, a pathology primarily caused by the inability of adipose tissue to store fatty acids, also induces hepatic steatosis and hyperinsulinemia. Interestingly, 25.3% of individuals with NAFLD are lean, suggesting that NAFLD prevalence increases independently of obesity and genetic lipodystrophy [3].

Hyperinsulinemia is deemed a major driver of simple steatosis, as hyperinsulinemia can develop in obese, lean, and lipodistophic individuals [3,4]. However, prolonged fasting in normo-insulinemic healthy mice can also induce fatty liver, indicating that hepatic steatosis can occur independently of hyperinsulinemia. In normo-insulinemic fasting, hepatic steatosis is explained by white adipose tissue releasing more fatty acids and glycerol than needed for liver mitochondria to cover their demand for ATP. This fatty acid excess is stored in intrahepatic lipid droplets to prevent free fatty acid-mediated toxicity, while concurrently allowing hepatocytes to have a reservoir of fatty acids.

The requirement of mitochondrial function to oxidize fat, generate ketones, and increase hepatic glucose production, as well as to increase lipid synthesis and storage raises a simple question: Should we decrease or increase mitochondrial oxidative function in the liver to reverse hepatic steatosis and decrease hepatic glucose production? The answer is further complicated by the fact that mitochondrial respiration regulates intracellular signaling by releasing reactive oxygen species (ROS), particularly hydrogen peroxide (H_2_O_2_). At low concentrations, H_2_O_2_ produced by mitochondria can transduce a signal modulating the activity of multiple enzymes. These include kinases and phosphatases, such as protein tyrosine phosphatase 1B, that regulate transcriptional programs determining gluconeogenesis, lipogenesis, and insulin sensitivity [5,6]. In this context, how will a change in mitochondrial oxidative function impact H_2_O_2_-regulated mechanisms controlling glucose and lipid metabolism in hepatocytes?

In this article, we review recent studies to answer these questions and discuss new potential approaches that target mitochondria to combat NAFLD and hyperglycemia.

## 2 The role of mitochondrial oxidative function in NAFLD

### 2.1 Decreased mitochondrial oxidative function contributes to NAFLD

**Figure 1:**
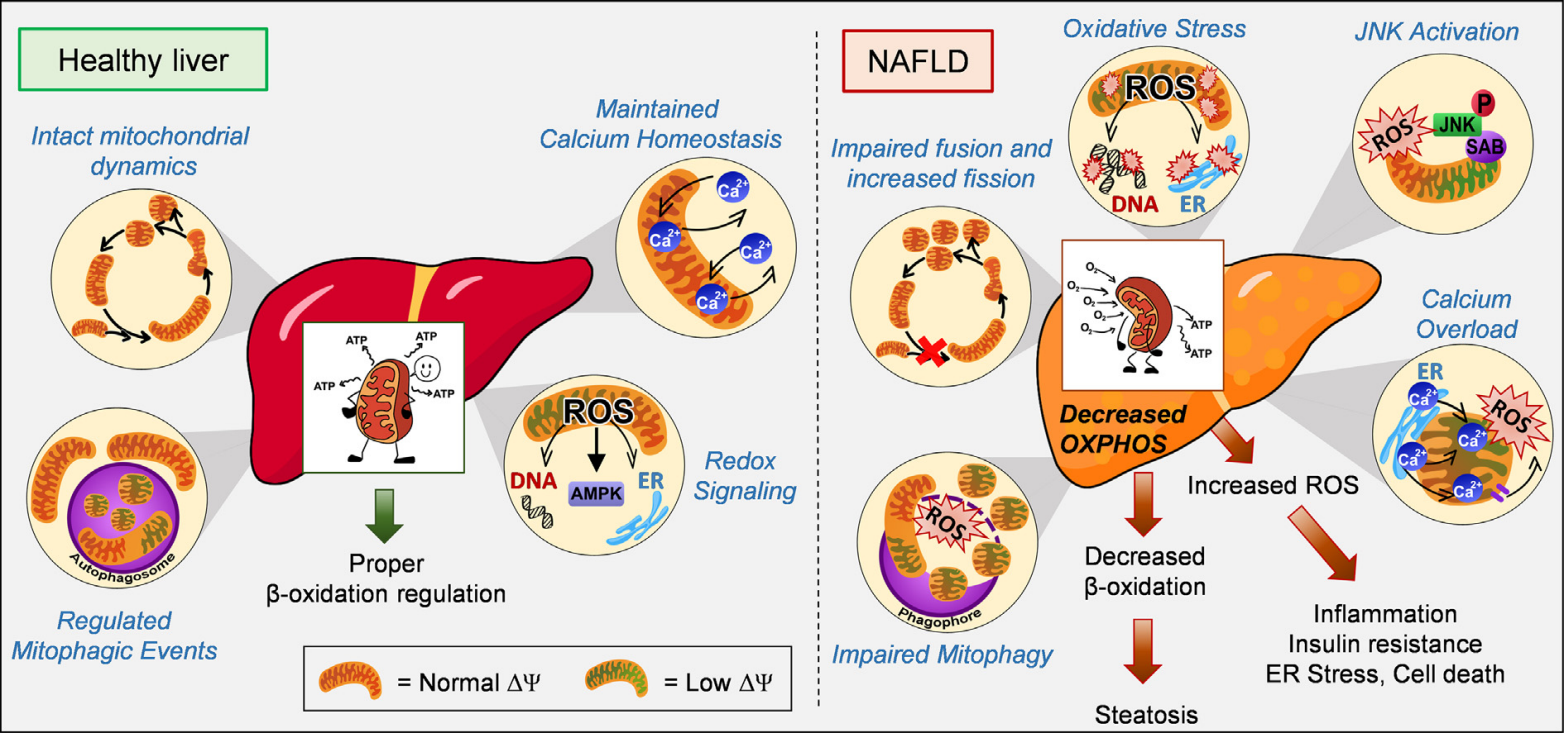
Mitochondria as a compromised friend in NAFLD. Mitochondria in hepatocytes oxidize fatty acids to produce ketone bodies, as well as ATP to cover glucose production during fasting. Mitochondria rely on diverse mechanisms to preserve their function including dynamics, redox signaling, mitophagy and calcium homeostasis. In contrast to a healthy liver, mitochondria in NAFLD were reported to be fragmented, overloaded with calcium, with decreased oxidative capacity and increased ROS production, which cause JNK activation. JNK activation itself can induce these same defects in mitochondrial function, constituting a feed-forward cycle of mitochondrial dysfunction. Mitochondrial dysfunction in NAFLD was also explained by defective mitophagy. The decrease in fatty acid oxidation caused by this compromise in mitochondrial function was deemed to induce fat accumulation in hepatocytes, while impairing insulin signaling. ER, endoplasmic reticulum; JNK, c-Jun NH2-terminal Kinase; SAB, SH3 homology associated BTK binding protein; Dy, mitochondrial membrane potential; ROS, reactive oxygen species.

A decrease in mitochondrial ATP-synthesizing respiration was observed in mouse models with simple steatosis or NASH, concluding that fat accumulates in the liver because the mitochondria cannot oxidize enough fatty acids (Figure 1). Accordingly, genetic and diet-induced obesity lowers mitochondrial respiration measured in isolated mouse hepatocytes [7,8]. Mechanistically, high-fat diet feeding causes an excessive increase in calcium uptake by liver mitochondria that disrupts respiration and elevates ROS production [7]. Defective respiration and thus decreased fat consumption was attributed as a major factor driving steatosis, while mitochondrial ROS-mediated c-Jun-N terminal kinase (JNK) activation was responsible for the disruption of insulin signaling that promotes hyperglycemia [7]. Overall, this study showed that cellular control of mitochondrial respiration by calcium released from the ER is a major contributor to mitochondrial dysfunction in simple steatosis.

The upregulation of hepatic ceramide (Cer) synthesis caused by high-fat diet feeding was shown to decrease mitochondrial respiration and drive both simple steatosis and NASH in mice. Indeed, the sphingolipid Cer16:0 itself, synthesized by the enzyme CERS6, had a direct action decreasing mitochondrial fat oxidation in hepatocytes from mice with simple steatosis. In addition, Cer16:0 decreased hepatic insulin signaling in mice, leading to hyperglycemia [9]. Higher hepatic ceramide content was also associated with decreased mitochondrial oxidative capacity in human NASH [10]. The inhibitory effect on mitochondrial function induced by ceramides was attributed to mitochondrial fragmentation, more specifically by the direct interaction of ceramides with the mitochondrial and peroxisomal fission/fragmentation factor (Mff) [11].

#### 2.1.1 The role of mitochondrial fragmentation in the decrease in mitochondrial oxidative function observed in simple steatosis and NASH

Mitochondrial fragmentation refers to the process by which long mitochondria segregate into smaller units, which can be induced by inhibiting fusion or activating fission. While mitochondrial fragmentation can amplify apoptosis [12], it is unclear why mitochondrial fragmentation mediated by Mff decreases mitochondrial fat oxidation and promotes steatosis [11,13].

Mitochondrial fission executed by Drp1 and Mff is a physiological process that occurs in healthy cells. Fission executed by Drp1 was shown to increase fatty acid oxidation in adipocytes and to be recruited in other tissues as an adaptive mechanism counteracting nutrient excess [14,15]. Accordingly, fragmentation of mitochondria in hepatocytes by deleting the mitochondrial fusion protein Mfn1 increased mitochondrial oxidative function in mice with simple steatosis [16].

Furthermore, fragmentation does not only allow mitochondrial oxidative function to adapt to changes in ATP demand and nutrient availability. Indeed, mitochondrial fragmentation allows autophagy to remove damaged mitochondria, a process called mitophagy [14,17]. Autophagy and thus mitophagy require intact lysosomal function, which is achieved by preserving their acidity (low pH). Remarkably, fatty acids and the concurrent increase in ceramides elevate lysosomal pH in pancreatic beta cells and the liver, which disrupts the digestive function of lysosomes and halts autophagic flux [[18], [19], [20]]. Consequently, lysosomal dysfunction causes the accumulation of damaged mitochondria by impairing mitophagy, with lysosomal dysfunction even directly causing mitochondrial damage via the release of lysosomal components [21,22]. It is possible that lysosomal dysfunction and disruption in mitophagy, rather than mitochondrial fragmentation per se, are the main mechanisms behind ceramides decreasing mitochondrial function.

Of note, mitochondrial fragmentation induced by hepatocyte-specific deletion of the mitochondrial fusion protein mitofusin 2 (Mfn2) exacerbates NAFLD progression, inflammation and hyperglycemia in high-fat diet-fed mice [23,24]. These data could be interpreted as evidence supporting the view that mitochondrial fragmentation drives steatosis and NASH by decreasing fat oxidation. However, Mfn2 plays additional roles beyond counteracting fragmentation, explaining the opposite effects caused by Mfn2 *vs* Mfn1 deletion. Mfn2, but not Mfn1, regulates ER mitochondria contacts [25] and transfers phospholipid intermediates between mitochondria and ER, facilitating phospholipid synthesis [23]. Thus, this role of Mfn2 promoting phospholipid synthesis could explain how Mfn2 aided the formation of new autophagosomal membranes that are generated from ER phospholipids [26].

Major metabolic consequences of deleting Mfn2 in hepatocytes are decreased mitochondrial respiration, reduced fat oxidation, elevated ROS production and JNK activation [23,24]. Interestingly, the exacerbation of NASH and the decrease in hepatic fat oxidation in mice induced by Mfn2 deletion were explained by ER stress, not by mitochondrial fragmentation [16,23,24]. Mechanistically, Mfn2 deletion reduced the transfer of phospholipid intermediates between mitochondria and ER, causing PERK hyperactivation. Prevention of ER stress in Mfn2 KO livers decreased NASH and insulin resistance and reversed the decrease in fat oxidation induced by Mfn2 deletion [23,24,27]. Importantly, preventing ER stress in Mfn2 KO livers did not reverse hepatic steatosis, despite increasing fat oxidation [23]. Thus, it seems that the decrease in fat oxidation in Mfn2 KO livers would only contribute to inflammation. Hence, the mechanism by which Mfn2 deletion promotes hepatic steatosis is unclear. A potential mechanism will be discussed in Section 3.

#### 2.1.2 JNK can induce mitochondrial fragmentation, decrease fat oxidation, and elevate mitochondrial ROS in simple steatosis and NASH

Some reports support that decreased mitochondrial fat oxidation and elevated ROS production in simple steatosis and NASH are initiated by the activation of c-Jun-N terminal kinase (JNK), which can phosphorylate mitochondrial proteins. Exposing primary hepatocytes to high levels of fatty acids activates JNK to phosphorylate the mitochondrial protein SAB. SAB phosphorylation results in elevated mitochondrial ROS production and decreased respiration [28]. Moreover, JNK phosphorylates Mfn2 to activate its degradation [29] and blocks PPARa-mediated upregulation of mitochondrial fat oxidation in livers from obese mice [30]. Accordingly, hepatocyte-specific deletion of JNK1/2 protects obese mice from hepatic steatosis, enlarges mitochondria (phenocopying Mfn2 gain of function), and elevates mitochondrial fatty acid oxidation via transcriptional upregulation of mitochondrial proteins [30].

A current therapeutic approach to treat NAFLD by promoting mitochondrial fat oxidation is using PPARa and d-dual agonists (NCT02704403). Preclinical data supporting this approach include data showing that PPARa-mediated increases in fat oxidation occur without promoting glucose production in humans. In mice, PPARa agonism properly mimics fasting, as mitochondrial fatty acid oxidation (FAO) is upregulated to match the ATP demand imposed by gluconeogenesis. This human-mouse difference suggests that PPARa activation in humans might drive an uncharacterized ATP-demanding process different from gluconeogenesis. Mitochondria will only increase FAO if ATP demand increases, as ATP synthase controls respiration rates in coupled mitochondria.

Another option would be that PPARa increases the expression of a mitochondrial uncoupler in humans. Consequently, more fatty acids would be oxidized to produce the same amount of ATP. In agreement with this hypothesis, pharmacological approaches uncoupling mitochondria selectively in hepatocytes from rodents and non-human primates protect from hepatic steatosis [31,32]. This hepatocyte-restricted uncoupler also improved insulin signaling in the liver and muscle, the latter by reducing hepatic lipid export to muscle [31,32]. Hence, the ATP sink that allows PPARa activation to promote fat oxidation without elevating gluconeogenesis in humans remains to be identified.

The transition from simple steatosis to NASH is associated with a decrease in mitochondrial oxidative capacity. This decrease supports that the upregulation of mitochondrial fat oxidation might also be a feasible therapeutic approach to treat NASH. However, the decrease in mitochondrial function in NASH has been attributed to mitochondrial damage caused by ceramides [10]. Thus, repairing the damage and/or eliminating ceramides, rather than stimulating the damaged mitochondria that are already there, might be a safer approach to treat NASH.

#### 2.1.3 Defects in mitophagy contribute to reduced oxidative capacity in NAFLD

Mitochondrial depolarization, which can be caused by decreased respiratory function, is the major event targeting mitochondria to autophagy (mitophagy). As mitochondria from livers with NASH showed decreased respiratory function, one could conclude that mitophagy is not effectively recruited in NASH. In other words, the mechanisms that repair and/or remove damaged mitochondria need to fail or be dormant to allow the presence of a large number of damaged mitochondria.

Accordingly, a recent study showed that hepatocyte-specific deletion of Parkin, an E3 ubiquitin ligase selectively targeting damaged mitochondria to mitophagy, exacerbates fatty liver disease and insulin resistance in high-fat diet-fed mice [33]. The exacerbation of NAFLD induced by Parkin deletion supports the view that mitophagy is still active in simple steatosis. Thus, the additional decrease in mitochondrial respiration observed in NASH could be triggered by the acquisition of an additional defect in mitophagy. In agreement with this conclusion, the decrease in Mfn2 activity induced by a greater activation of JNK caused by inflammation could contribute to impaired mitophagy, as Mfn2 aids the formation of autophagosomes needed for mitophagy [23,26]. These studies suggest that removing damaged mitochondria by activating mitophagy could be a promising approach to counteract simple steatosis and NASH.

In agreement with this conclusion, the in vitro activation of the glucagon receptor (GCGR) in primary hepatocytes isolated from mice with NASH was sufficient to increase mitophagy and mitochondrial oxidative function [8]. The activation of the glucagon receptor was achieved with a dual agonist of GLP1R and GCGR, called cotadutide. Accordingly, in vivo cotadutide treatments of mice fed an amylin diet, a model of diet-induced NASH markedly improved liver histopathology [8]. The ability of cotadutite to increase mitophagy in a hepatocyte-autonomous manner supports that mitophagy is silenced in NASH rather than irreversibly suppressed. The rescue of mitophagy and mitochondrial function induced by cotadutide depends on PKA activity [8]. Interestingly, PKA induces mitochondrial fragmentation by increasing Drp1 recruitment to mitochondria, where it binds to Mff, while Drp1-mediated fragmentation is required for mitophagy [14,15,17,34]. Thus, cotadutide possibly causes PKA-dependent fragmentation to facilitate mitophagy as a defense mechanism against simple steatosis and NASH. Overall, the PKA-dependent benefits of cotadutide action further support that fragmentation is a physiological process and not a synonym of mitochondrial dysfunction. Cotadutide is currently in clinical trials for NASH (NCT04019561).

### 2.2 Mitochondrial oxidative function is not impaired in NAFLD

Not all studies have shown that mitochondrial oxidative function is decreased or impaired in simple steatosis and NASH. Indeed, no defects were observed in the respiratory function of liver mitochondria isolated from ob/ob mice with hepatic steatosis [35]. Furthermore, the capacity of isolated liver mitochondria to oxidize fatty acids was even increased in ob/ob mice [35]. However, and in marked contrast to the increase in FAO observed in isolated mitochondria, a decrease in mitochondrial FAO was observed in perfused livers and isolated hepatocytes from obese rats with fatty liver [36].

An integrated interpretation of these seemingly contradictory findings is that fatty liver does not result from primary damage to mitochondria, but is rather caused by an alteration in how hepatocytes regulate and control mitochondrial FAO [37]. This decrease in FAO observed in perfused livers with steatosis was concluded to be a result for the inhibition of carnitine palmitoyl-transferase 1 (CPT1) [35,37]. Accordingly, the rate-limiting step of mitochondrial FAO is executed by CPT1, which catalyzes the entry of long-chain fatty acids into mitochondria. An endogenous mechanism to block CPT1 activity is elevated glycolysis, which promotes the synthesis of the CPT1 inhibitor malonyl-CoA. Consequently, it could be hypothesized that obesity associated with hyperglycemia causes steatosis by blocking CPT1 and FAO in the liver via excessive production of malonyl-CoA. This malonyl-CoA would be diluted and washed by the isolation procedure, explaining the increase in FAO when measured in isolated mitochondria. Consequently, reducing malonyl-CoA synthesis or decreasing CPT1 sensitivity to malonyl-CoA-mediated inhibition was deemed as a strategy to prevent steatosis. In agreement with this view, the treatment of obese mice with PPARa agonists decreased the ability of malonyl-CoA to inhibit CPT1 in isolated liver mitochondria [38]. The mechanism(s) by which PPARa agonists decrease the sensitivity of CPT1 to malonyl-CoA-mediated inhibition is unclear. However, studying these mechanisms of CPT1 sensitivity to malonyl-CoA could be harnessed to reverse hepatic steatosis.

**Figure 2:**
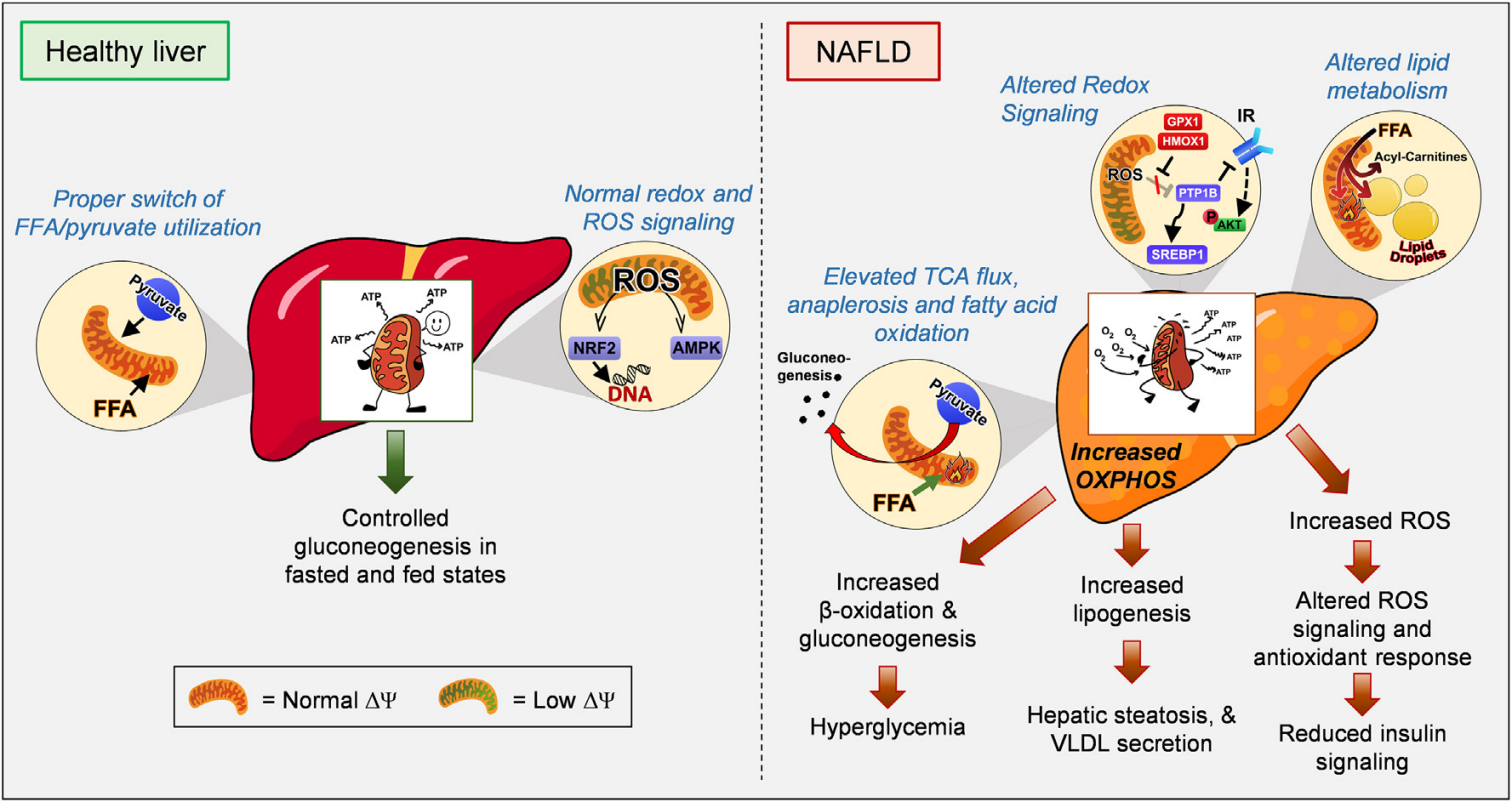
Mitochondria as a foe in NAFLD. As the liver supplies glucose, ketone bodies and lipids to other organs, proper control of hepatic gluconeogenesis and lipid metabolism in the fasted and fed state is essential. NAFLD is associated with increases in hepatic glucose production and lipid synthesis/storage in part due to higher glucose and lipid supply. The elevation in gluconeogenesis and lipid storage increase mitochondrial ATP demand, explaining the increase in mitochondrial oxidative capacity reported in simple steatosis and even in NASH. Interestingly, fatty liver is not only associated with increased mitochondrial fat oxidation, which normally fuels glucose production and ketogenesis, but it also increases TCA cycle flux. Further, it was proposed that increased oxidative function of mitochondria elevates ROS production, which can underpin inflammation, impaired insulin signaling and cell death. Evidence suggests that increased TCA cycle flux and impaired ketogenesis cause hepatic steatosis and hyperglycemia, supporting that restoration of mitochondrial fuel preference can be a therapeutic target for NAFLD. IR, insulin receptor; GPX1, glutathione peroxidase 1; HMOX1, heme oxygenase-1; PTP1B, protein-tyrosine phosphatase 1B; SREBP1-c, Sterol regulatory element-binding transcription factor 1-c; FFA, free fatty acids; VLDL, very low densitity lipoprotein.

Developments in non-invasive metabolite labeling and tracing in the liver allowed the quantification of FAO and mitochondrial fluxes in intact livers from mice and humans [39,40]. These non-invasive studies support that the suppression of FAO observed in isolated hepatocytes and perfused livers with steatosis does not occur in vivo (Figure 2). Accordingly, humans with simple steatosis and insulin resistance show elevated mitochondrial FAO and respiratory function when measured non-invasively in vivo and even ex vivo after isolating mitochondria from liver biopsies [[39], [40], [41]]. Preservation of higher respiratory capacity after isolating mitochondria demonstrates that the composition of mitochondria is changed by NAFLD. From these data, one can conclude that higher FAO and mitochondrial function observed in NAFLD is not just a consequence of a change in the cellular control of FAO and mitochondrial respiration [39]. Further, this change in mitochondrial composition might reflect an adaptation to the chronic increase in gluconeogenesis and intrahepatic lipid handling induced by NAFLD, which increase the demand for mitochondrial ATP and TCA cycle intermediates (Figure 2). This mitochondrial remodeling might also prevent free fatty acid-mediated toxicity in hepatocytes.

Increased mitochondrial FAO in the liver from patients with simple steatosis and insulin resistance agrees with the hypothesis presented by McGarry entitled “What if Minkowski had been Ageusic?” [42]. McGarry argued that increased FAO in the liver could be a major driver of hyperglycemia in diabetes, as FAO supports hepatic glucose production. Thus, if Minkowski could not have tasted glucose in the urine, but smelled the ketones in the breath resulting from FAO, diabetes treatments would have aimed to block FAO and restore lipid metabolism. Supporting McGarry's hypothesis, short-term treatment of type 2 diabetic humans and obese rodents with etomoxir, a drug blocking CPT1 and thus FAO, showed positive effects on insulin sensitivity, decreased glycemia, and even circulating lipids [43,44]. However, longer-term treatments with etomoxir exacerbated steatosis and hyperglycemia in obese rodents [43], demonstrating that the benefits of blocking FAO on glycemic control are only transient. It is possible that chronic etomoxir treatments could mimic the systemic fatty acid spillover characteristic of lipodystrophy and obesity. This spillover would outcompete the beneficial effect of etomoxir decreasing hepatic glucose production. Supporting this view, the increase in acylcarnitine intermediates due to incomplete fatty acid oxidation is associated with NAFLD [45,46] and these intermediates were shown to contribute to insulin resistance in muscle [47].

The increase in respiratory capacity and FAO are not the only changes observed in mitochondria from mice and humans with NAFLD. There is also a change in the proportion of fuels that support mitochondrial respiration (Figure 2). In humans and mice with fatty liver, mitochondrial pyruvate oxidation and TCA cycle flux are elevated in the fasted state, while ketogenesis does not increase [40]. As FAO is a major source of ketones, the lack of ketosis seemed contradictory to elevated FAO observed in obese humans with insulin resistance [41]. This apparent contradiction was reconciled by data showing that FAO-derived acetyl-CoA, which is normally used for ketogenesis, is diverted to the TCA cycle in simple steatosis [48]. Indeed, in healthy liver mitochondria, FAO results in a decrease in TCA cycle flux and citrate synthase activity, which facilitates ketogenesis by diverting acetyl-CoA away from citrate synthase [49]. Thus, a key pathogenic mechanism in NAFLD could be the acquired ability of mitochondria to sustain high TCA fluxes concurrently with high FAO rates. It is possible that a primary defect in the machinery synthesizing ketones is driving the elevation in TCA flux in NAFLD by making more acetyl-CoA available to the TCA cycle [50].

In agreement with high TCA fluxes participating in NAFLD pathogenesis, decreasing TCA cycle fluxes by deleting the mitochondrial pyruvate importer MPC1 diminishes both hepatic glucose production and inflammation in high-fat diet-fed mice [51,52]. Hepatitis driven by high MPC1 activity was attributed to an increase in mitochondrial ROS production concurrent with a decrease in antioxidant capacity. The increase in mitochondrial ROS production was deemed to result from the perennial elevation in mitochondrial respiration caused by the constant anabolic needs of fatty livers [52,53]. However, hepatic steatosis was unchanged in liver-specific MPC1 or MPC2 KO mice fed a high-fat diet [51,52,54,55]. In this regard, MPC deletion is expected to decrease *de novo* fatty acid synthesis that is fueled by citrate synthesized from mitochondrial pyruvate (see Section 1 and the next paragraph) and would not decrease dietary fatty acid esterification [56]. Thus, preserved steatosis in high-fat diet-fed (HFD) MPC KO mice is in agreement with HFD feeding mostly inducing steatosis by increasing dietary fatty acid esterification into intrahepatic TG [56].

In contrast to HFD-induced hepatic steatosis in mice, up to 38% of hepatic TG accumulated in humans with fatty liver stems from de novo synthesized fatty acids [57]. This relatively high percentage indicates the possibility that MPC inhibition could efficiently protect humans from steatosis by decreasing *de novo* fatty acid synthesis. The reason is that mitochondrial acetyl-CoA generated from pyruvate oxidation fuels citrate and malonyl-CoA synthesis (see Section 1). Accordingly, pharmacological inhibition of the enzymes responsible for malonyl-CoA synthesis, acetyl-CoA carboxylases (ACC1 and ACC2), reversed hepatic TG accumulation in humans [58]. Remarkably, although ACC inhibition resulted in elevated mitochondrial FAO and ketogenesis, as expected by unleashing CPT1 activity, blocking ACC activity markedly increased plasma lipid levels [58].

The increase in plasma lipids was explained by the upregulation in hepatic triglyceride synthesis (TG) selectively directed toward VLDL assembly and excretion. Elevated VLDL assembly was attributed to the transcriptional upregulation of the rate-limiting enzyme in hepatic TG synthesis, GPAT1, as a result of increased SREBP-1c activity [58]. However, transcriptional upregulation of GPAT1 alone cannot explain how some fatty acids escape mitochondrial FAO and are selectively directed toward VLDL assembly. Thus, elevated circulating levels of TG and VLDL by ACC inhibition strongly support the existence of a pool of fatty acids that escape from fat oxidizing mitochondria and are exclusively destined to VLDL synthesis. We present a hypothesis on how some fatty acids can escape mitochondria oxidation in Section 3, proposing a new mechanism.

**Figure 3:**
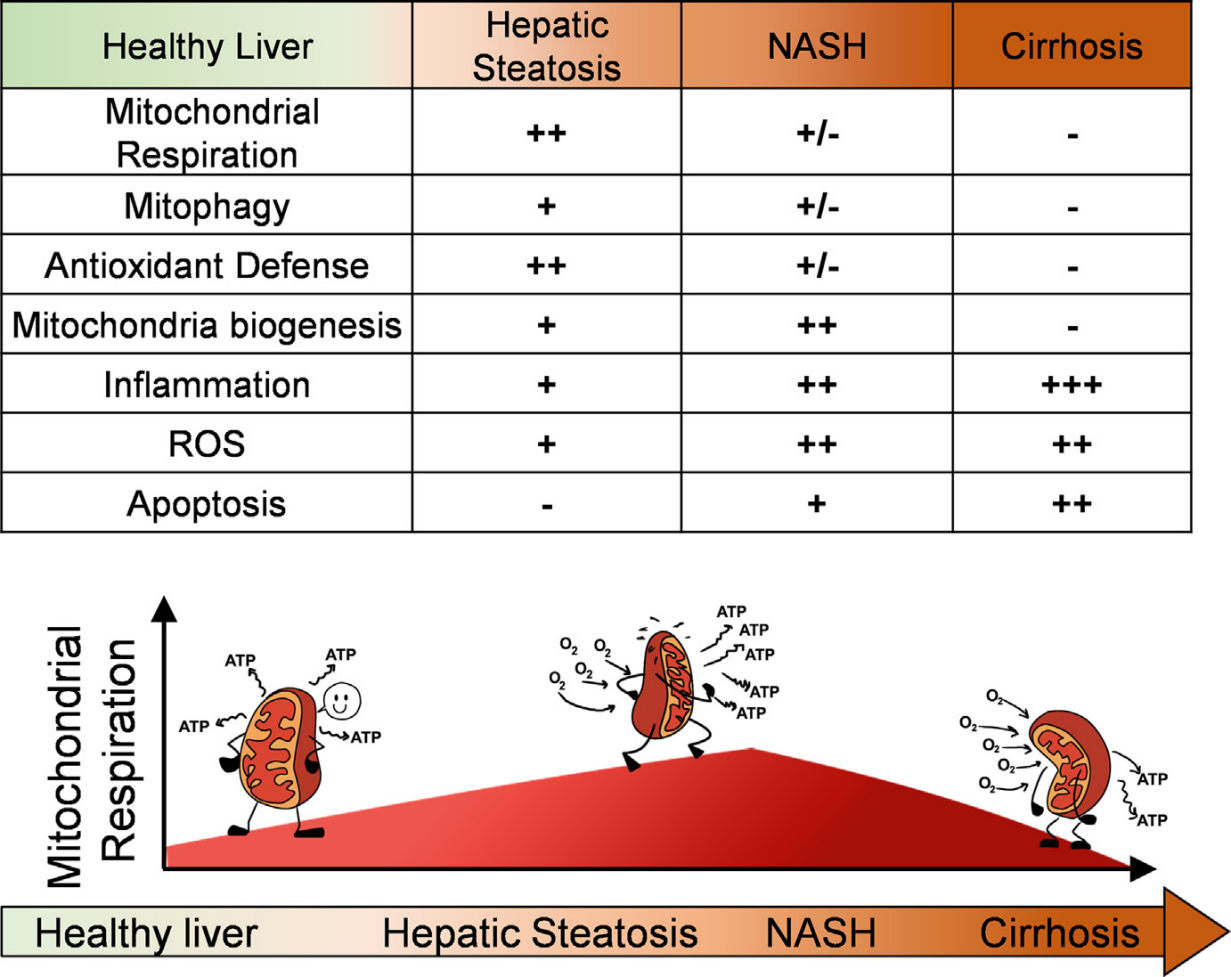
Mitochondrial states throughout the progression of NAFLD. In the early stages of NAFLD, namely simple steatosis, adaptative mechanisms occur to compensate for the increase in fuel availability and anabolism: mitochondrial respiration increases due to higher substrate availability and increased ATP demand, which will increase ROS prodcution, activate mitochondrial biogenesis and antioxidant responses. As hepatocytes store more lipids and reach full storage capacity, free-fatty acid mediated toxicity impairs mitophagy and damages mitochondria. In the transition to NASH, mitochondrial function is decreased and ROS is further increased, which were deemed to be responsible for higher inflammation and cell death characteristic of NASH. However, some mouse models with antioxidant enzymes selectively deleted in hepatocytes are protected from NASH, questioning whether the increase in ROS observed in simple steatosis and NASH contributes to the disease. In certain cases, NASH with fibrosis will progress to cirrhosis, meaning that hepatocytes will be replaced with cell types with fewer and dysfunctional mitochondria, contributing to the decline in liver oxidative function. FFA, free fatty acids; VLDL, very low densitity lipoprotein; TG, triglycerides

The transition from simple steatosis to NASH is associated with a reduction in total oxidative capacity measured in isolated mitochondria and liver homogenates [39,59]. Perez-Carreras et al. showed that the individual activities of each mitochondrial OXPHOS complex were lower in NASH patients than in lean controls, particularly ATP synthase, while Koliaki et al. reported that NASH patients showed higher mitochondrial respiratory capacity than lean controls. The decrease in mitochondrial oxidative capacity in NASH can have two interpretations: i) it is an acquired defect in the transition to NASH that further exacerbates steatosis and ROS production (Figure 3) or ii) it is an adaptive mechanism to inflammation and uncontrolled steatosis, aiming to limit anabolism and ROS production from mitochondrial OXPHOS. The reduction in OXPHOS as an adaptive mechanism can explain why mitochondrial mass is preserved in patients with NASH or, in other words, why mitophagy does not eliminate these mitochondria deemed as dysfunctional based on lower OXPHOS.

If the reduction in OXPHOS is an adaptive mechanism, one could interpret the specific reduction in ATP synthase and increase in proton leak as evidence of regulated mitochondrial remodeling caused by NASH rather than damage. Proton leak together with decreased complex V allow mitochondria to escape the limitation in fat oxidation imposed by ATP demand, while impeding an additional increase in ROS by limiting electron transport chain activity. This premise is supported by NASH patients still showing higher oxidative capacity that lean controls, but lower than in simple steatosis [39].

## 3 Functionally distinct subpopulations of mitochondria may explain contradictory observations of mitochondrial function in NAFLD

Mitochondrial dynamics not only refer to the processes causing fragmentation of mitochondria into smaller units or their elongation to form interconnected networks. Mitochondrial dynamics also refer to mitochondrial motility, spatial distribution, and their contacts with other organelles. Electron microscopy studies in the late 1950s demonstrated that the nutritional status regulated the architecture of mitochondria in the liver and exocrine pancreas from rodents [60,61]. Fasting expanded lipid droplets and brought mitochondria in close contact with those lipid droplets in hepatocytes and pancreatic acinar cells [60,61]. When these images were generated in the 1950s, two conflicting hypotheses were already presented: one favored that mitochondria surrounding lipid droplets were synthesizing fat and/or esterifying imported fatty acids into lipid droplets, which explained fasting-induced lipid droplet expansion [60,61]. The other stated that mitochondria close to lipid droplets oxidized fatty acids released by lipid droplets, as FAO increased with fasting [60,61]. To date, the exact role of mitochondria bound to lipid droplets remains unanswered in hepatocytes. We will discuss how mitochondrial dynamics can clarify the conundrum of the role of mitochondrial oxidative function in hepatic steatosis. Particularly, why measurements of mitochondrial function in intact livers and isolated mitochondria both reveal increased oxidative function induced by steatosis, while measurements of primary hepatocytes and perfused livers showed the opposite.

### 3.1 The role of peridroplet mitochondria in NAFLD

Our study of brown adipose tissue (BAT) demonstrated that mitochondria bound to lipid droplets, or peridroplet mitochondria (PDM), can promote TG synthesis and expand lipid droplets [62]. The functional demonstration that PDM participated in TG synthesis was possible due to genetic manipulation of the lipid droplet protein perilipin 5 (PLIN5). The expansion of lipid droplets mediated by PLIN5 was severely diminished when PLIN5's ability to recruit mitochondria was abrogated [62]. Thus, PLIN5 not only increases the size and number of lipid droplets by blocking lipolysis [63], but also expands lipid droplets by recruiting mitochondria to elevate TG synthesis [62]. We showed that ATP production by PDM was required by PLIN5 to increase TG synthesis [62]. Moreover, we demonstrated that PDM had a higher capacity to oxidize pyruvate and synthesize citrate [62].

These characteristics of PDM in BAT have a strong resemblance to the changes in mitochondrial function observed when hepatic lipogenesis is elevated. As a result, we expect that increasing the interaction of hepatocyte mitochondria to lipid droplets would promote steatosis. Published studies characterizing the role of PLIN5 in liver support this expectation. Indeed, hepatic PLIN5 gain of function increased the number of lipid droplets and induced steatosis in hepatocytes as we showed in brown adipocytes [63,64]. Remarkably, hepatic steatosis induced by PLIN5 did not cause hyperglycemia or hepatic inflammation and even protected from high-fat diet-induced insulin resistance [64]. However, deleting PLIN5 in hepatocytes resulted in PPARa activation, which elevated mitochondrial FAO and prevented hepatic steatosis at the expense of causing hepatic damage and inflammation [63]. Thus, PLIN5 loss-of-function in the liver resembles cold-induced activation of thermogenic FAO in BAT, which decreases the number of peridroplet mitochondria by an unclear mechanism.

**Figure 4:**
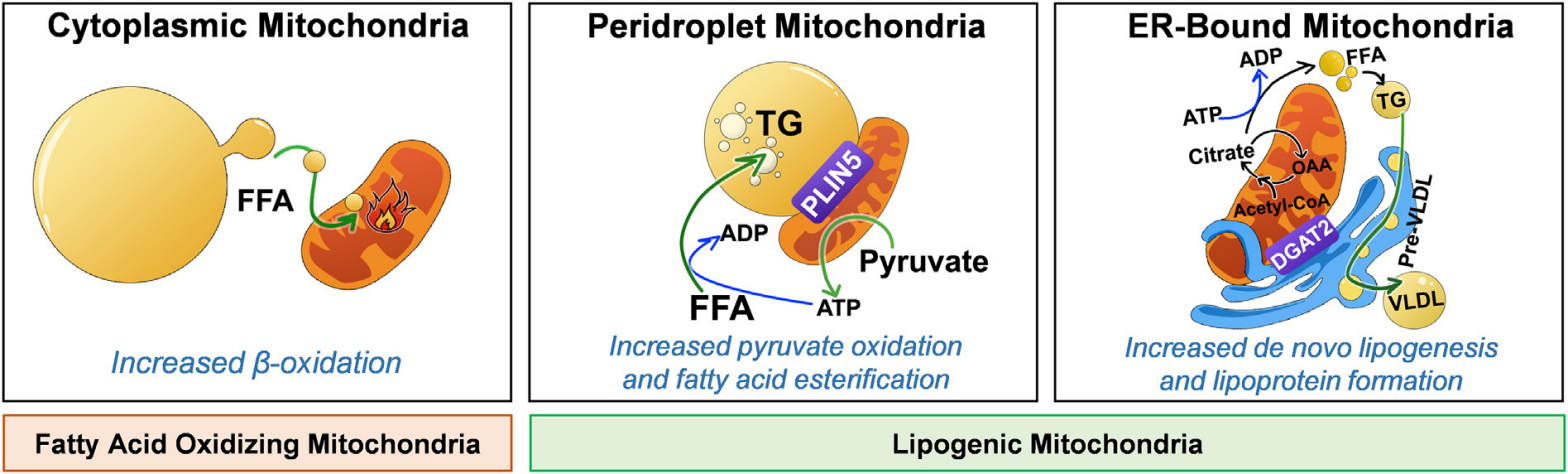
Proposed model of three distinct mitochondrial populations in hepatocytes. Mitochondria attached to different organelles were shown to have distinct functions, demonstrating that not all mitochondria in the same cell are homogeneous. This concept supports that: 1) Different mitochondria can be specialized in a specific task: some mitochondria in hepatocytes can be specialized in synthesizing lipids, while other mitochondria can oxidize lipids. 2) Localizing mitochondria close to their targeted organelles or compartments have the advantage to exchange metabolites or molecules more efficiently. The functional segregation of mitochondria can be determined by their anchorage to specific organelles, which prevents motility and thus fusion between the different subpopulations. Based partly on our previous work [61], we propose the existence of 3 mitochondrial populations in hepatocytes: 1) Cytosolic mitochondria, which are responsible for fatty acid oxidation, ketone bodies production and ureagenesis to support glucose production; 2) mitochondria attached to lipid droplets, namely peridroplet mitochondria (PDM), which promote the esterification of fatty acids into triglycerides and; 3) the ER-anchored mitochondria, which are responsible for fatty acid synthesis, lipoprotein assembly and excretion. VLDL, very low densitity lipoprotein; PLIN5, perlipin 5; DGAT2, Diacylglycerol O-Acyltransferase 2; ER, endoplasmic reticulum; OAA, oxaloacetate.

In summary, hepatic PLIN5 traps the dietary excess of fatty acids in lipid droplets, protecting hepatic from insulin resistance and damage induced by high-fat diet feeding. Furthermore, PLIN5 loss-of-function in hepatocytes demonstrates that PPARa activation and elevated mitochondrial FAO will not be beneficial if the capacity of hepatocytes to sequester fatty acid excess into lipid droplets is impaired. Consequently, one could envision that a therapeutic strategy for NAFLD would require a concurrent increase in the capacity of PDM to sequester fatty acids and cytosolic mitochondria oxidizing fat (Figure 4).

### 3.2 Three distinct mitochondrial subpopulations could regulate hepatic steatosis in opposite ways in NAFLD

Our BAT study showed that at least two segregated populations of mitochondria exist, which we called peridroplet mitochondria (PDM) and cytosolic mitochondria (CM) [62]. The segregation of PDM from CM was achieved by the physical interaction of mitochondria with lipid droplets. Mechanistically, anchoring mitochondria to lipid droplets decreased their motility, which impeded their ability to fuse with other mitochondria and thus prevented homogenizing mitochondrial content [62]. Furthermore, this segregation caused by anchoring mitochondria to lipid droplets allowed PDM to be functionally different, increasing their oxidative function fueling ATP synthesis, while harboring a higher capacity to oxidize pyruvate and synthesize citrate [62]. Therefore, we propose that hepatocytes also harbor a first subpopulation of segregated mitochondria similar to BAT PDM, which would be specialized to support dietary fatty acid esterification into TG. Hepatic PDM could be defined by mitochondria anchored to lipid droplets via PLIN5, a lipid droplet protein that binds mitochondria in multiple tissues, or via other uncharacterized proteins.

In addition to dietary fatty acid esterification into TG, hepatocytes can synthesize new fatty acids and assemble lipoproteins in the ER. These lipoproteins carry TG, cholesterol, and phospholipids, allowing their export from hepatocytes to plasma. We propose that a second subpopulation of segregated mitochondria, ER-anchored mitochondria, would have their CPT1 activity inhibited and would support both lipoprotein assembly and de novo fatty acid synthesis. We further propose that these ER mitochondria would have a limited capacity to execute FAO, similar to PDM. Finally, a third distinct subpopulation of mitochondria would exist, namely cytosolic mitochondria (CM), which would be specialized in oxidizing fatty acids (Figure 4).

The existence of ER-anchored mitochondria can explain how a pool of fatty acids escapes FAO and can be selectively directed toward lipoprotein assembly (VLDL). This subpopulation of specialized mitochondria can explain why ACC inhibition reduces intrahepatic TG content in mice, while increasing VLDL secretion [58]. The segregation of mitochondria caused by their interaction with the ER allows these ER mitochondria to have different protein compositions and/or post-translational modifications, inhibiting their capacity to oxidize fatty acids. An interesting property could be that these mitochondria selectively carry CPT1B, an isoform of CPT1 that is highly sensitive to malonyl-CoA inhibition. As hyperinsulinemia promotes lipogenesis, hyperinsulinemia might explain why ER mitochondria contacts increase in mice with insulin resistance and simple steatosis [7].

PLIN5 gain of function in the liver causes cytosolic lipid droplet expansion without increasing VLDL export and plasma lipids [64]. This published study supports that PLIN5 and potentially PDM do not increase *de novo* lipogenesis or the assembly of lipoproteins in the ER. We thus propose that, instead of PLIN5, one of the important mediators that segregate mitochondria to generate ER-anchored lipogenic mitochondria is DGAT2 [65]. DGAT2 catalyzes the last step of TG synthesis and is located in the mitochondria-associated membranes (MAM) of the ER. Furthermore, DGAT2 itself can promote the association of mitochondria to the ER, and DGAT2 deletion decreases steatosis and hypertriglyceridemia [66].

Mfn2 is also known to participate in ER mitochondria contacts [25], and Mfn2 deletion in hepatocytes causes hepatic steatosis [23,24,27]. Thus, it is possible that steatosis induced by Mfn2 KO is caused by dysfunctional and/or unstable ER mitochondria contacts, limiting the ability of mitochondria to support VLDL assembly. Limited or impaired transfer of phospholipid precursors and ATP between the mitochondria and ER could affect both lipoprotein assembly as well as fatty acid esterification into TG in the ER. Therefore, impairment in VLDL assembly caused by Mfn2 deletion could cause the re-routing of fatty acids and TG into cytosolic lipid droplets. This specific role of Mfn2 in VLDL assembly could explain why the reversal of ER stress, inflammation, decreased FAO, and insulin resistance in liver-specific Mfn2 KO mice does not decrease hepatic steatosis [12,18,19].

The hypothetical scenario that ER mitochondria and PDM in the liver are functionally separated from cytosolic mitochondria can explain the divergence in the effects reported on mitochondrial oxidative function in NAFLD. Segregating mitochondria into three different populations inside one hepatocyte means that the oxidative function of each population can be completely independent. The functional independence of each segregated population can explain why TCA cycle fluxes and FAO rates measured *in* *vivo* concurrently increase in NAFLD, despite FAO and TCA being competing fluxes that can cancel each other in isolated mitochondria [49].

Moreover, approaches used to measure mitochondrial function might favor detecting one mitochondrial population vs the others. We believe that isolating primary hepatocytes and liver perfusion might reduce the function of mitochondria oxidizing fatty acids and PDM. The absence of a proper provision for extracellular fatty acids and hormones (i.e., glucagon) in respirometry assays of primary hepatocytes and perfused livers could reduce the function of these mitochondrial subpopulations that are more predominant in NAFLD. It is also possible that cell culture or perfusion media could induce some mitochondrial remodeling in livers and primary hepatocytes, which is not observed in freshly isolated mitochondria from the liver. Thus, this mitochondrial remodeling could explain why functional studies in isolated mitochondria from NAFLD, despite seemingly being the less physiologically relevant approaches, are more consistent with mitochondria oxidative function measured *in* *vivo*.

The establishment of methods to separate these 3 mitochondrial populations will be essential to demonstrate their existence and specialization in hepatocytes. One approach could be using PLIN5 to pull down PDM and DGAT2 to pull down ER-anchored mitochondria, with mitochondria that do not bind to PLIN5 and DGAT2 being cytosolic mitochondria that oxidize fatty acids. We could then compare their capacity to synthesize citrate and oxidize fatty acids, as well as their sensitivity to malonyl-CoA-mediated inhibition of FAO. We expect that cytosolic mitochondria will be the population with the highest FAO capacity and lowest sensitivity to malonyl-CoA-mediated FAO inhibition. However, lipogenic mitochondria (PDM and ER mitochondria) have higher citrate synthase activity, higher sensitivity to malonyl-CoA-mediated FAO inhibition, and lower FAO capacity. Another approach would be to identify specific mitochondrial proteins acting as biomarkers for each population. Once identified, these biomarkers could be used to visualize them using microscopy or quantify their relative abundance. However, it is difficult to anticipate the existence of an exclusive protein that defines each population. We would rather expect a differential enrichment of certain proteins.

## 4 The role of H_2_O_2_ released by mitochondria and endogenous antioxidants in NAFLD

### 4.1 Mitochondrial H_2_O_2_ as a signal transducer and modulator of mitochondrial oxidative function

Reactive oxygen species (ROS) include different oxidants such as superoxide, hydrogen peroxide (H_2_O_2_), and hydroxyl radicals. The main ROS produced by healthy mitochondria is superoxide [67], which is an unstable anion that mitochondrial and cytosolic dismutases transform into H_2_O_2_. Then H_2_O_2_ is reduced to water by different systems, including peroxiredoxins (Prx) and mitochondrial glutathione peroxidases (Gpx). Although mitochondria can eliminate H_2_O_2_, mitochondria from healthy livers still release H_2_O_2_. Since H_2_O_2_ is potentially harmful, why do mitochondria release H_2_O_2_ in health?

H_2_O_2_ is the major ROS that acts as a signaling molecule, as H_2_O_2_ is sufficiently stable and lipophilic to cross mitochondrial membranes. Mechanistically, H_2_O_2_ can directly or indirectly oxidize thiols from exposed cysteines of different proteins, resulting in a reversible or irreversible change in their function. Thiol oxidation generates sulfenic acid (-SOH), which can form disulfide bonds with nearby cysteines (-S-S-) or undergo further oxidation to sulfinic (-SO_2_H) or sulfonic (-SO_3_H) acids. Except for sulfonic and to a lesser degree sulfinic acid, which can also lead to protein degradation, thiol oxidation is reversible. Reversibility allows cysteine oxidation to act as a signal, which might explain why mitochondrial proteins are preferentially oxidized on cysteines [68]. In addition, H_2_O_2_-mediated oxidation is necessary for cellular and mitochondrial adaptations to different stresses [69]. As a result, H_2_O_2_ generated in the mitochondria is not only relevant to directly modulate mitochondrial function by oxidizing its proteins, but also to transduce signals that activate a cellular response.

**Figure 5:**
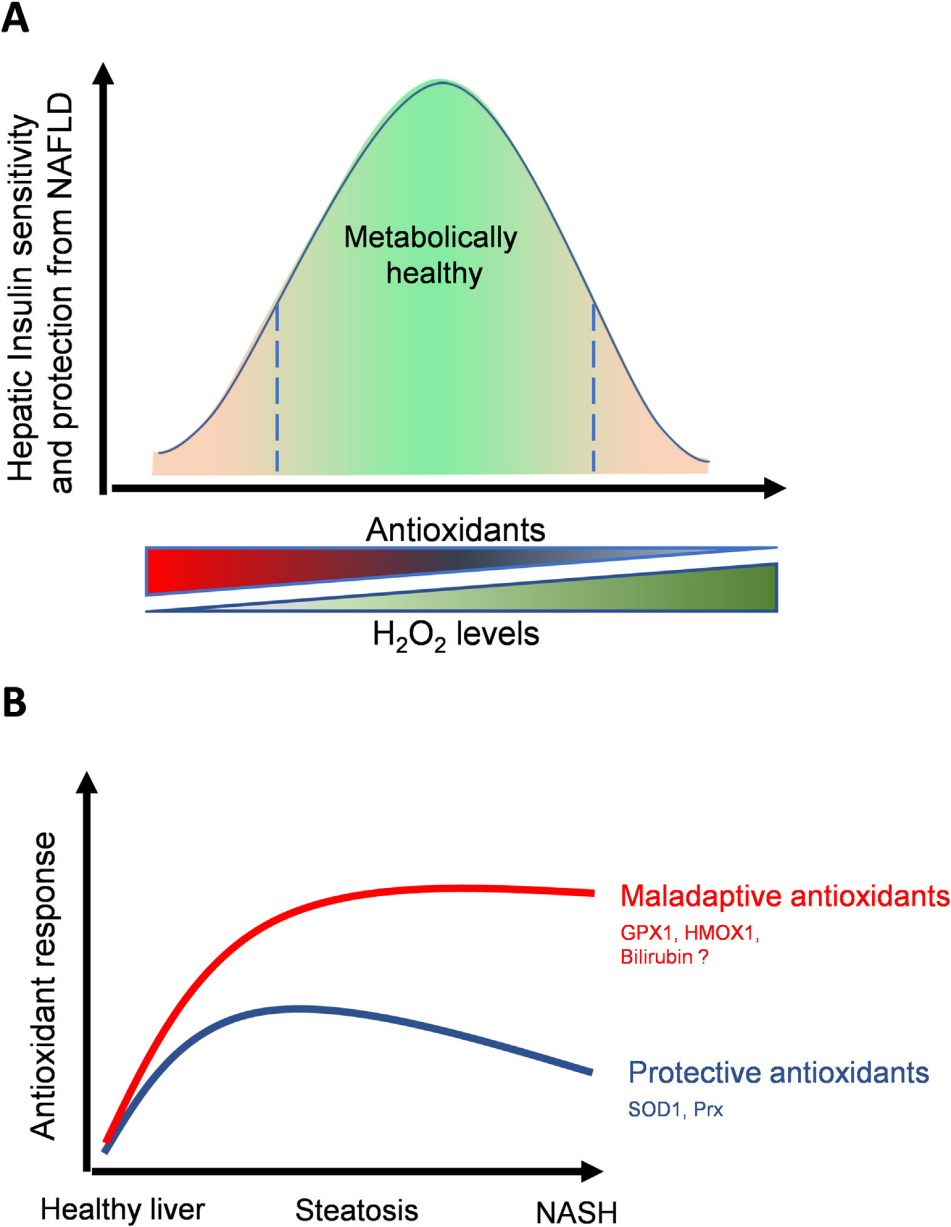
Heterogenous antioxidant systems reveal a spectrum of protective and maladaptive antioxidant responses in NAFLD. **A)** The essential role of reactive oxygen species (ROS) as a signaling molecule for physiological processes demonstrated that impairment of ROS signaling caused by excessive antioxidant activity can be as deleterious as uncontrolled ROS production. Indeed, insulin resistance and NAFLD have been reported to be caused by excessive ROS production or excessive antioxidant activity. As these are two opposite processes driving the same disease, two options exist to explain these findings: i) In some individuals, increased antioxidant function rather than excessive ROS drives pathogenesis. ii) The different subcellular localization of different antioxidant systems can explain the concurrency of increased ROS in one compartment and increased antioxidant function in another compartment, both contributing to pathogenesis. **B)** Identified maladaptive antioxidants that contribute to NAFLD by eliminating mitochondrial H_2_O_2_ are GPX1, HMOX1, and potentially bilirubin. On the other hand, superoxide dismutase 1, which transforms superoxide to H_2_O_2_, and Prx, which removes H_2_O_2_, were shown to protect from NAFLD. The divergent and opposite role of these different antioxidant systems in NAFLD development demonstrates that H_2_O_2_ can have opposite roles depending on where it is generated and removed. Bilirubin is a lipophilic antioxidant, whose production can be increased when HMOX1 activity is upregulated. Bilirubin has actions on the mitochondrial electron transport chain as well, but the role of these actions of bilirubin in the mitochondria to NAFLD development have not been characterized yet. GPX1, glutathione peroxidase 1; HMOX1, heme oxygenase 1; Prx, Peroxiredoxin, SOD1, Superoxide dismutase 1.

Despite this regulatory and signaling role of H_2_O_2_, a large defect in H_2_O_2_ scavenging capacity or excessive H_2_O_2_ production leads to uncontrolled oxidative stress and cell death. As a result, H_2_O_2_ is also considered a toxic byproduct that exacerbates age-related diseases. A simplified model integrating both views is that low levels of H_2_O_2_ production are beneficial and essential for normal physiology, while excessive H_2_O_2_ production that is not balanced by antioxidants causes disease and death. However, this simplified model does not consider that proteins removing or producing H_2_O_2_ are largely compartmentalized, with their activity controlled by independent systems. Consequently, it is possible that in the same disease and same cells, one compartment might show excessive H_2_O_2_ production, while the other might demonstrate excessive antioxidant activity, with both events driving pathogenesis (Figure 5).

### 4.2 Redox stress and mitochondrial H_2_O_2_ production in NAFLD

Increased mitochondrial H_2_O_2_ production in hepatocytes occurs in patients with simple steatosis and NASH. In simple steatosis, increased mitochondrial H_2_O_2_ production reflects an adaptive mechanism, concurrent to elevated mitochondrial function, ATP synthesis, and oxidative modification of lipids and proteins [39]. Therefore, these data on mitochondrial H_2_O_2_ in simple steatosis demonstrate that oxidative modifications, rather than damaging mitochondria, are signals preserving the increase in anabolism fueled by higher mitochondrial oxidative function.

Koliaki et al. demonstrated that NASH patients showed an additional increase in mitochondrial H_2_O_2_ production and a decrease in their oxidative function compared to patients with simple steatosis [39]. This additional increase in mitochondrial H_2_O_2_ in NASH was concurrent with a decrease in catalase activity. These two processes were deemed responsible for the exacerbation of oxidative damage in hepatic lipids, proteins, and DNA in NASH [39]. Thus, if increased mitochondrial H_2_O_2_ production contributed to NASH pathogenesis, the expectation would be that inactivating enzymes removing H_2_O_2_ in the mitochondrial matrix and cytosol would unequivocally exacerbate NASH. However, two mouse models and human studies support the opposite outcome, namely that removing antioxidants does not induce NASH and even protects from simple steatosis [5,70].

In this regard, hepatocyte-specific deletion of glutathione peroxidase 1 (GPX1), an enzyme located in the mitochondrial matrix and cytosol that eliminates H_2_O_2_, protected mice from diet-induced NASH and hyperglycemia [70]. The increase in H_2_O_2_'s actions induced by GPX1 deletion led to the oxidative inactivation of protein-tyrosine phosphatase 1B (PTP1B). Hyperactivation of PTP1B induces insulin resistance and steatosis, as PTP1B dephosphorylates the insulin receptor and increases SREBP-1c activity [71,72]. The PTP1B catalytic domain contains a key cysteine residue whose oxidation blocks its phosphatase activity and can even lead to PTP1B proteolysis. As a result, PTP1B inactivation by H_2_O_2_ improved insulin signaling and protected from steatosis [73]. Remarkably, deleting heme oxygenase 1 (HMOX1) in hepatocytes protected mice from NAFLD and hyperglycemia by increasing H_2_O_2_-mediated PTP1B inactivation [5]. While the mechanism by which GPX1 deletion increased H_2_O_2_ actions in hepatocytes was obvious, the mechanism by which HMOX1 deletion increased H_2_O_2_ actions was harder to predict. The source of beneficial H_2_O_2_ actions induced by HMOX1 deletion was demonstrated to be mitochondria [5], but the exact mechanism responsible for these mitochondrial changes remains unclear.

Heme degradation by HMOX1 generates biliverdin, which is rapidly transformed into bilirubin. Bilirubin decreases respiration and ATP synthesis in isolated mitochondria [74] and diminishes superoxide and H_2_O_2_ levels [75,76]. In addition, bilirubin is detected inside mitochondria of cell lines, but its role in mitochondrial physiology in hepatocytes is yet to be determined [77]. Consequently, the exact role of mitochondrial bilirubin in the development of NAFLD remains unknown. However, given the benefits of deleting HMOX1 in hepatocytes from mice with steatosis, bilirubin could be an example of a maladaptive antioxidant promoting NAFLD and insulin resistance. Of note, bilirubin is a lipophilic antioxidant and most reports showing that antioxidants prevent NAFLD characterized hydrophilic antioxidants [78]. As lipophilic molecules can be localized in different compartments compared to hydrophilic molecules, lipophilic antioxidants might have different signaling actions *vs* hydrophylic antioxidants [78]. Thus, exploring and identifying endogenous lipophilic antioxidants acting on mitochondria might provide novel insights into NAFLD development.

AMPK is another player modulating and responding to mitochondrial function. AMPK is a kinase that increasesmitochondrial function by elevating mitochondrial biogenesis and mitophagy [79]. Hepatocyte-specific expression of constitutively active AMPK protected mice from hepatic steatosis and inflammation, while improving systemic glucose homeostasis, decreasing fat mass, elevating FAO and increasing autophagic flux [80,81]. While AMPK can be activated by changes in ROS [82], no studies have evaluated whether AMPK activation by mitochondrial H_2_O_2_ release can be exploited to treat simple steatosis and NASH. A known protein kinase increasing AMPK activity is the ataxia-telangiectasia mutated kinase (ATM), which is activated by mitochondrial ROS. ATM enhances mitophagy, antioxidant function, and different responses to DNA damage. Interestingly, patients with ataxia-telangiectasia caused by ATM inactivation show higher risk for NAFLD [83]. Ataxia-telangiectasia patients are more susceptible to developing mitochondrial dysfunction, which might explain their increased risk of developing NAFLD [84].

The beneficial actions of mitochondrial H_2_O_2_ counteracting NAFLD might explain the failure of different clinical trials testing ROS scavengers to treat NAFLD. While pre-clinical studies in mice showed that untargeted ROS scavengers (resveratrol or other polyphenols) protected from simple steatosis, studies in humans led to conflicting results [85]. Polyphenols showed beneficial effects on NASH in some clinical trials, while others showed no improvement, and one trial even found that high doses of resveratrol damaged liver function [86,87]. This latter trial supports that excessive and untargeted removal of H_2_O_2_ can become as deleterious as excessive H_2_O_2_ production.

The GSH:GSSG and Cys:CysS pairs are crucial regulators of the cellular redox state, with their ratio determining ROS levels and H_2_O_2_-mediated actions. The redox potential of these pairs is the key parameter that determines their ability to decrease ROS. Remarkably, these pairs show markedly different redox potentials depending on the subcellular compartment where they are located [88,89]. Subcellular heterogeneity in redox potential shows that ROS levels and H_2_O_2_-mediated actions in each organelle and compartment can be independently regulated. As a result, each organelle and compartment can show different susceptibilities to the detrimental and beneficial actions of H_2_O_2_. With this heterogeneity, it is hard to conceive that NAFLD will cause a concurrent and similar imbalance in H_2_O_2_ production and antioxidant function in all subcellular compartments. A better understanding of the compartmentalization of ROS production and antioxidant systems is thus required to determine the exact involvement of mitochondrial H_2_O_2_ in NAFLD.

## 5 Current and hypothetical therapies targeting liver mitochondria to treat NAFLD and hyperglycemia

Mitochondrial function is maintained through a complex interplay between cellular ATP demand, nutrient availability, the ability of mitochondria to communicate energetic and redox stress, and the response of mitochondria to energetic imbalances. NAFLD is primarily a consequence of an energetic, redox, and hormonal imbalance in hepatocytes, leading to excessive exposure of mitochondria to nutrients relative to their ATP demand. Moreover, mitochondria in NAFLD perennially facilitate anabolism and lipid synthesis when it is not needed. Therefore, one way to cure NAFLD could be to reduce the anabolic activity of mitochondria or alternatively decrease nutrient loads by activating mitochondrial function to eliminate these nutrients. We list current approaches that target mitochondria to treat NAFLD and propose novel approaches based on our hypothesis of mitochondrial segregation in hepatocytes.

Modulating mitophagy could decrease lipogenesis in the liver by targeting lipogenic mitochondria for degradation. Indeed, preclinical studies suggested that mitochondrial quality control and mitophagy are reduced in NAFLD, with the restoration of mitophagy preventing or reversing some features of simple steatosis and NASH [[90], [91], [92]]. One could hypothesize that lipogenic mitochondria accumulate in simple steatosis and NASH because hyperinsulinemia and hyperlipidemia suppress their elimination by mitophagy. Agreeing with this hypothesis, treatment with a natural molecule that restores mitophagy, urolithin A [93], improves glucose homeostasis and protects mice from hepatic steatosis [94]. Moreover, current drugs improving steatosis and NASH in humans, such as metformin and liraglutide, increase mitophagy in rodent models of simple steatosis and NASH [95,96]. Cotadutide, which targets GLP1R and GCGR and improves NASH, also enhances mitophagy [8]. Of note, cotadutide mimics glucagon action, implying that mitophagy of lipogenic mitochondria might be a new mechanism by which glucagon decreases lipogenesis. Understanding whether hepatic GCGR activation can selectively target lipogenic mitochondria for mitophagy would resolve the conundrum on how eliminating mitochondria, which oxidize fatty acids, can counteract steatosis.

AMPK activators could potentially eliminate lipogenic mitochondria and even replace them with fat-oxidizing mitochondria [97]. Alternatively, mitophagy can be improved by restoring lysosomal function, as hepatic lysosomal acidity is lower in NAFLD [18,98,99]. Lysosomes are essential for autophagy/mitophagy and can determine hepatic TG content [100]. Therapies to restore lysosomal function could represent a new strategy to attenuate NAFLD [101].

Another way to prevent steatosis without inducing hyperglycemia is by increasing FAO in mitochondria without increasing ATP synthesis (uncoupled respiration), which would prevent an increase in glucose production. Although mitochondrial uncouplers are cardiotoxic and their action in muscle causes malignant hyperthermia [102,103], a novel approach that delivered the active form of a mitochondrial uncoupler exclusively to hepatocytes showed a marked improvement in safety. This hepatic-specific uncoupler decreased hepatic steatosis and insulin resistance in obese rats, improved liver function, and prevented fibrosis in a rat model of NASH [32,104] and dysmetabolic non-human primates [31]. Other mild uncouplers delivered systemically, such as BAM15, improved systemic glucose homeostasis and insulin resistance, showing efficacy in mouse models of NAFLD [105].

As elevated oxidative damage is associated with all stages of NAFLD, different studies aimed to improve mitochondrial function using antioxidants. These trials tested mostly hydrophilic antioxidants and provided inconclusive data. Vitamin E is one of the few lipophilic antioxidants tested for NASH, which was also inconclusive. A meta-analysis showed that vitamin E ingestion was associated with decreased liver steatosis, but not improved fibrosis [106]. Moreover, vitamin E at high doses correlated with higher cancer and stroke incidence [107,108].

Dietary antioxidants that specifically decrease ROS in mitochondria showed benefits in mouse models of NAFLD, but their efficacy in treating NAFLD in humans has not been proven. One antioxidant tested in humans is silymarin, which is a mixture of flavonolignans with the major active component being silybinin [109]. Silymarin decreased oxidative damage in different subcellular compartments by decreasing mitochondrial respiratory chain activity [110]. Clinical trials revealed that treatment with silymarin decreased circulating AST and ALT levels and improved liver histology in participants with simple steatosis or NASH [111,112]. Systemic treatments with compounds scavenging ROS inside mitochondria, such as mitoQ or mito-tempo, protected rodents from metabolic syndrome, but evidence in humans is still missing [113,114]. Moreover, studies restricting the actions of these mitochondrial antioxidants to hepatocytes are also missing. Therefore, it is unknown if the benefits stemming from these antioxidant treatments are explained by their actions on the liver or in other tissues.

While protection from NAFLD induced by hepatocyte-specific deletion of GPX1 and HMOX1 in mice is a proof of concept that excessive antioxidant function can promote NAFLD, no therapeutic strategies and trials aimed to decrease antioxidant activity in human livers. As a result, targeting maladaptive and redundant antioxidant systems is a largely unexplored therapeutic avenue in NAFLD. It would be interesting to determine whether some of the current NAFLD treatments can decrease the expression of certain antioxidant systems. Strong candidates as maladaptive antioxidants would be among those induced by inflammation and decreased by GLP1R-GCGR agonism.

Furthermore, regulating mitochondrial dynamics and architecture could be a new therapeutic strategy to combat NAFLD. Enhancing the attachment of mitochondria to lipid droplets could protect from fatty acid-mediated toxicity during feeding. However, promoting detachment of mitochondria during fasting could enhance fat oxidation and elimination. This strategy would restore the ability of liver to handle lipids and prevent inflammation associated with lipotoxicity.

Overall, we conclude that novel therapeutic approaches to treat NAFLD can be provided by: i) methods limiting mitochondrial-driven synthesis of lipids and glucose, ii) removing maladapted mitochondria by mitophagy, or iii) decreasing the action of maladaptive and redundant antioxidant systems.
